# Evaluation of the Nutritional Composition and Microbiological Quality of Sorghum (*Sorghum bicolor* (L.) Moench)

**DOI:** 10.3390/foods14234079

**Published:** 2025-11-27

**Authors:** Angel Angelov, Ivan Rangelov, Mariana Petkova, Rosen Chochkov, Stefan Shilev, Velitchka Gotcheva

**Affiliations:** 1 Center of Competence AgriFood Systems and Bioeconomy, 4000 Plovdiv, Bulgaria; 2 Index 11 JSC, 4000 Plovdiv, Bulgaria; rangelovivan21@gmail.com; 3 Department of Microbiology and Environmental Biotechnologies, Agricultural University of Plovdiv, 4000 Plovdiv, Bulgaria; marandonova@gmail.com (M.P.); stefan.shilev@au-plovdiv.bg (S.S.); 4 Department of Technology of Cereals, Fodder, Bread and Confectionary Products, University of Food Technologies, 4000 Plovdiv, Bulgaria; r_chochkov@uft-plovdiv.bg; 5 Department of Microbiology and Biotechnology, University of Food Technologies, 4000 Plovdiv, Bulgaria; gotcheva_v@uft-bio.com

**Keywords:** sorghum hybrids, nutritional composition, antioxidant capacity, lactic acid bacteria, yeasts, microbial profiling, functional foods

## Abstract

Sorghum (*Sorghum bicolor* (L.) Moench) is increasingly recognized as a sustainable crop due to its adaptability to challenging environmental conditions and its nutritional potential. The present study aimed to characterize the nutritional composition and native microbial species associated with three sorghum hybrids cultivated in Bulgaria. Crude protein was 9.37–10.42%, total carbohydrate content was between 87.4 and 89.6%, and crude fat content was in the range of 3.84–4.9%. Linoleic acid was the predominant fatty acid in all hybrids, accounting for 44.9% to 48.0% of total lipids. Quinic acid emerged as the dominant organic acid in all hybrids, with the highest concentration of 729.37 mg/100 g. The microbiological assessment focused on lactic acid bacteria (LAB) and yeasts. Microbial isolates were subjected to molecular identification through 16S rRNA gene and ITS region sequencing. The predominant LAB species included *Levilactobacillus brevis*, *Lactiplantibacillus plantarum*, *Lactiplantibacillus pentosus*, *Pediococcus acidilactici*, and *Pediococcus pentosaceus*, while most of the yeast isolates belonged to *Saccharomyces cerevisiae*. Phylogenetic analysis indicated substantial intraspecies variation, particularly within LAB strains, suggesting the presence of unique genotypic traits. These findings contribute to a better understanding of sorghum’s nutritional value and endogenous microbiota and open opportunities for developing sorghum-based functional products.

## 1. Introduction

Sorghum (*Sorghum bicolor* (L.) Moench) is a cereal crop of considerable agronomic and nutritional value that is particularly well-suited to arid and semi-arid environments [1]. Native to Northeastern Africa and South Asia, sorghum is characterized by its exceptional tolerance to abiotic stressors such as prolonged drought, elevated temperatures, and low soil fertility. These adaptive traits have positioned sorghum as a pivotal crop in climate-resilient agricultural systems, supporting food security for over 500 million people worldwide. Sorghum is used to make some indigenous foods—injera, flatbread, non-alcoholic and alcoholic beverages, gluten-free breads and cereals, pasta, molasses, animal feed, as well as bioethanol, and biodegradable construction materials [2,3,4,5].

In recent years, due to its high drought resistance and plasticity, grain sorghum has been increasingly used in Bulgaria to obtain a stable yield of feed grain during the frequent extreme droughts. Scientific research is concentrated in two research organisations: the Institute of Forage Crops, Pleven, and the Agricultural Institute, Shumen, which run breeding and agronomy projects for the use of sorghum for feed purposes [6]. Market data are pushing farmers and companies to introduce sorghum as a diversification strategy, and in the year 2023, sorghum was cultivated on 30,000 da, with a total of 8000 t produced and an average yield of 267 kg/da. The main challenges are related to the introduction of new hybrids, improving the market structure, and technologies for processing sorghum into bioethanol, food and bioproducts [7].

From a nutritional perspective, sorghum is a naturally gluten-free grain, rich in complex carbohydrates, proteins (8–18%), dietary fiber, and essential micronutrients, including iron, zinc, and magnesium. Furthermore, it contains a diverse array of phytochemicals, such as phenolic acids, flavonoids, and anthocyanins, which contribute to its antioxidant capacity and potential health-promoting effects [3]. Unlike maize flour, sorghum does not present odor, which is a desirable feature for use in food products. It has been proven safe for people with celiac disease, but the literature on its use in gluten-free foods is relatively scarce compared to that on corn, teff, and rice [8,9].

The grain’s lipid profile is dominated by unsaturated fatty acids—primarily linoleic and oleic acids, known for their cardio-protective properties [10]. Its carbohydrate matrix, composed of slowly digestible starches and resistant starch fractions, supports glycemic regulation, making it a favorable dietary option for individuals with metabolic disorders such as diabetes, as well as those with gluten intolerance [11,12]. Furthermore, sorghum is rich in thiamine and riboflavin, integral to metabolic functions, along with high levels of niacin that improve digestion and skin health. It also comprises significant amounts of essential minerals, including phosphorous, magnesium, iron, copper, zinc, calcium, and potassium [13]. Also, sorghum’s unique composition of antioxidants is noteworthy; unlike conventional cereals such as wheat, maize, and rice, sorghum grains are a source of antioxidants due to phenolic compounds and 3-deoxy anthocyanins [14]. Beyond its nutritional composition, sorghum contributes to soil health through its extensive root system and interaction with beneficial rhizosphere microbiota, playing a role in nutrient cycling and carbon sequestration within agroecosystems [15,16,17].

The relationship between nutritional composition and microbial diversity is based on the natural symbiosis between sorghum and its associated microbial communities at all stages of plant and grain development. Understanding this relationship is the key to the possibilities to enhance nutrient acquisition, improve resilience to environmental stress, suppress diseases, and sustainably increase sorghum yields [18]. The type of microbial association varies according to the growing region and is heavily influenced by environmental conditions such as drought, rainfall, temperature, and sunlight, the harvesting and processing equipment, unsanitary handling, and poor storage conditions. Microbial contaminants are presented by bacteria, fungi, and other microorganisms [19,20,21]. However, the grain harbors endogenous microbial communities—particularly lactic acid bacteria (LAB) and yeast—that may exert beneficial functions [22]. These microorganisms are integral to natural fermentation processes, contributing to food preservation, flavor development, and the production of functional foods with probiotic potential [23,24]. Tank’s results indicated that moisture content, amino acid nitrogen, reducing sugar, residual starch, and total free amino acids were identified as the driving factors of the fungal and bacterial variation. The dominant microbial species were *Saccharomyces cerevisiae*, *Acetobacter pasteurianus*, and *Lactobacillus helveticus*, the last two being the predominant bacteria observed at the end of fermentation in glutinous sorghum and non-glutinous red sorghum, respectively. Moisture content and reducing sugar had a more significant impact on the microorganisms in glutinous sorghum, while amino acid nitrogen, total free amino acids, and residual starch were the main driving factors driving the microbial community in non-glutinous red sorghum [20]. Prior studies have highlighted the presence of such beneficial microbial taxa in traditional Bulgarian products, underscoring their biotechnological relevance and potential for health-promoting applications [25,26]. Studies on species diversity in sorghum grains play an important role in understanding the subsequent food fermentation process as well as in producing standardized, high-quality end products [27,28,29]. 16S rRNA gene-based methods are widely used to identify genetic relationships among bacteria [30]. With regard to yeast, the identification of new isolates to the species level is achieved via DNA sequencing and analysis of the ITS-5.8S-ITS2 region because of the high level of the interspecific sequence variability of ITS [31,32]. These tools enable the exploration of microbiomes in non-traditional substrates like sorghum, potentially unlocking strains with probiotic, antifungal, or technological functionality.

The present study aimed to evaluate the nutritional profile and native microbial communities associated with three sorghum hybrids cultivated under agroecological conditions in Bulgaria. By combining culture-based isolation techniques with molecular identification methods, the diversity of lactic acid bacteria and yeasts was characterized, with particular emphasis on their potential applicability in the development of functional, microbiota-enriched cereal-based food products.

## 2. Materials and Methods

### 2.1. Raw Materials

Grains of the sorghum hybrids Zealandia, Arabesk, and Kalatur (Lidea, 31700 Mondonville, France) were collected from Paskalevo village, Dobrich region, Bulgaria (GPS coordinates: 43°37′15.3″ N 27°51′19.1″ E). The grain bulks of the three hybrids were separately dried in industrial silos to a moisture content of approximately 12%. Samples from the bulk batches were taken and transported to the Department of Biotechnology, UFT, Plovdiv, Bulgaria. The samples were stored at room temperature for a week until analysis. All chemicals, reagents, and solvents were of analytical grade and purchased from FOT Ltd. (Sofia, Bulgaria), unless otherwise specified.

### 2.2. Chemical Compositions

***The crude fat, crude protein, and moisture content*** of the sorghum hybrids were determined according to American Association of Cereal Chemists (AACC) methods 30–10, 46–12, and 44–15, respectively [33]. Protein content was calculated with a protein factor of 6.25.

The ***total carbohydrate content*** of the grain samples was analyzed with the phenol-sulfuric acid method using glucose for the calibration curve. The dried sample was milled in a laboratory grinder, and an appropriate amount was further solubilized in 72% (*w*/*w*) H_2_SO_4_ (1 h, 30 °C), and after dilution with water to 1 M H_2_SO_4_, hydrolysis was performed for 3 h at 100 °C. The obtained hydrolysate was used as a sample for analysis. The absorbance was measured at 490 nm [34].

***The free phenolic fraction*** from representative samples (1 g) was extracted with 15 mL of solvent containing 75% ethanol at room temperature on a magnetic stirrer for 1 h. Then, the sample was centrifuged (6000× *g*, 20 min), and the supernatant was collected. Polyphenol content was determined according to the method of Singleton and Rossi with the Folin–Ciocalteu reagent. Gallic acid (10–200 µg/mL) was used as a standard [35].

The ***organic acids*** were extracted and analyzed according to Ognyanov et al. [36]. Then, the organic acids underwent UHPLC determination on a Nexera-i LC2040C Plus system (Shimadzu Corporation, Kyoto, Japan) equipped with a UV detector (210 nm). The separation was conducted on a Shim-pack GIST C18 (5 µm, 250 mm × 4.6 mm) column at 25 °C and a flow rate of 1.0 mL/min. Freeze-dried samples (approximately 1 g) were extracted with 30 mL of distilled water for 1 h at 30 °C in a shaking water bath. Following centrifugation at 6000× *g*, the supernatants were analyzed for organic acids using UHPLC. The sample was eluted isocratically using a 25 mM solution of K_2_HPO_4_ in water as a mobile phase (pH 2.4). The concentration of each organic acid in the sample was calculated using a calibration curve obtained using different concentrations for each acid. The peak corresponding to different acids was confirmed through a comparison of the retention time with that of the standards.

The ***free sugars*** were extracted and analyzed according to Ognyanov et al. [36]. Then, of the free sugars underwent chromatographic separation and quantification on a Nexera-i LC2040C Plus UHPLC system (Shimadzu Corporation, Kyoto, Japan), coupled with a Zorbax Carbohydrate column (4.6 mm × 150 mm, 5 μm) and Zorbax Reliance Cartridge guard-column operating at 35 °C. Approximately 1 g of each freeze-dried sample was extracted with 30 mL of distilled water for 1 h at 30 °C on a shaking thermostatic water bath. After centrifugation at 6000× *g*, the supernatants were used for the UHPLC analysis of sugars. The samples were injected (10 μL) and eluted with a mobile phase composed of a mixture of acetonitrile/H_2_O (80/20 *v*/*v*) at a flow rate of 1.0 mL/min. The eluate was monitored using a refractive index detector RID-20A (Shimadzu Corporation, Kyoto, Japan) (cell temperature: 40 °C). The concentration of sugars in the sample was deduced using a calibration curve built by plotting the peak area against different concentrations for each sugar. The different sugars in the sample were confirmed through a comparison of retention time with that of the standards.

***The oxygen radical absorbance capacity*** (ORAC) was measured according to [37] with some modifications [38]. Solutions of 2,2′-azobis (2-amidino-propane) dihydrochloride (AAPH), Fluorescein (FL), and trolox were prepared in a phosphate buffer (75 mmol/L, pH 7.4). Samples were diluted in the phosphate buffer as well. The reaction mixture (total volume 200 μL) contained FL (170 μL, final concentration 5.36 × 10^−8^ mol/L), AAPH (20 μL, final concentration 51.51 mmol/l), and the sample (10 μL). The FL solution and sample were incubated at 37 °C for 20 min directly in a microplate reader, and AAPH (dissolved in buffer at 37 °C) was added. The mixture was incubated for 30 s before the initial fluorescence was measured. After that, the fluorescence readings were taken at the end of every cycle (1 min) after shaking. For the blank, 10 μL of phosphate buffer was used instead of the extract. Trolox solutions (3.125; 6.25; 12.5; 25 and 50 μmol/L) were used to define the standard curve. ORAC was measured using a FLUOstar OPTIMA plate reader (BMG Labtech, Offenburg, Germany), using an excitation wavelength of 485 nm and emission wavelength of 520 nm. ORAC values were expressed in µmol тrolox equivalents (TE) per gram of dry weight (DW).

***The hydroxyl radical averting capacity (HORAC)*** was determined as described by Ou et al. [39]. Briefly, a hydrogen peroxide solution of 0.55 M was prepared in distilled water. Then, 4.6 mM Co(II) was prepared as follows: 15.7 mg of CoF_2_.4H_2_O and 20 mg of picolinic acid were dissolved in 20 mL of distilled water. Moreover, 170 μL of FL (60 nM, final concentration) and 10 µL of sample were incubated at 37 °C for 10 min directly in the FLUOstar plate reader. After incubation, 10 μL H_2_O_2_ (27.5 mM, final concentration) and 10 μL Co(II) (230 µM, final concentration) solutions were added. The initial fluorescence was measured, after which the readings were taken every minute after shaking. For the blank sample, phosphate-buffered solution was used. Then, 100, 200, 600, 800, and 1000 μM gallic acid solutions (in phosphate buffer 75 mM, pH = 7.4) were used to build the standard curve. Measurements were performed on a FLUOstar OPTIMA fluorometer (BMG LABTECH, Offenburg, Germany). An excitation wavelength of 485 nm and an emission wavelength of 520 nm were used. The results were expressed in µmol gallic acid equivalents (GAE) per gram of DW.

The ***fatty acid composition*** of oils was determined using the ISO gas chromatography method [40]. Fatty acid methyl esters (FAMEs) were obtained through the transesterification of the oils with 2% sulfuric acid in absolute methanol at 50 °C. The fatty acid composition was determined on an Agilent 8860 gas chromatograph (Agilent Technologies, Santa Clara, CA, USA) equipped with a DB Fast FAME capillary column (30 m × 0.25 mm × 0.25 μm (film thickness)) and a flame ionization detector. The temperature regime was as follows: 70 °C (1 min), increasing by 6 °C/min to 180 °C and by 5 °C/min to 250 °C; injector temperature—270 °C; detector temperature—300 °C. The carrier gas was nitrogen with a flow rate of 25 mL/min. Identification was performed by comparing the retention times of a standard 37-component mixture of FAMEs (Supelco, USA, 37 comp. FAME mix) subjected to GC under identical experimental conditions. The quantitative composition of the fatty acids was determined using the percentage ratio between the areas of the individual peaks in the chromatogram.

### 2.3. Microbiological Analysis

#### 2.3.1. Sample Preparation

A total of 0.3 g of sorghum grains was added over 5 mL of sterilized Tween 80 (0.01% (*v*/*v*) in a bottle containing 20 glass beads. The suspension was stirred for 2 min using a Vortex mixer. The suspension was diluted when necessary, and 0.1 mL aliquots were inoculated on specific culture media as described below.

#### 2.3.2. Total Viable Count, Lactic Acid Bacteria, and Yeast Enumeration and Isolation

For the ***total viable counts***, plate count agar (Merck KGaA, Darmstadt, Germany ) was used. The plates were kept in the incubator for 48 h, and the concentration of mesophilic microorganisms in the samples was quantified by counting the colony-forming units (CFU). MRS (Merck KGaA, Darmstadt, Germany) was used to determine the ***viable counts of LAB*** in the samples. The medium was supplemented with cycloheximide (0.1 g/L). The plates were incubated under anaerobic conditions (AnaeroGen, Oxoid Ltd., Hampshire, UK) at 37 °C for 48 h. From each medium, a number of colonies equal to the square root of the total number recorded in Petri dishes with 15 to 300 CFUs were randomly selected for isolation. The isolates were examined microscopically and tested via Gram staining and catalase reaction. Pure cultures were further obtained from the isolates that were Gram-positive, catalase-negative, nonmotile rods and cocci after sub-culturing in the respective liquid medium and streaking on agar media. Stock cultures were stored in Microbank™ vials (Pro-Lab Diagnostics Inc., Richmond Hill, ON, Canada) at 70 °C. The ***viable counts of yeasts*** in the samples were estimated on malt extract agar (MEA) (Merck KGaA, Darmstadt, Germany) supplemented with chloramphenicol (0.1 g/L) at 30 °C for 72 h. From each medium, a number of colonies equal to the square root of the total number recorded in Petri dishes with 15 to 300 CFUs were randomly selected for isolation. Morphological characterization of the yeast isolates was performed via microscopic analysis. The isolates were sub-cultured in malt extract broth and streaked onto the same agar media. Stock cultures were stored in Microbank™ vials (Pro-Lab Diagnostics Inc.) at −70 °C.

### 2.4. Molecular Identification of Lactic Acid Bacteria via 16S rRNA Gene Sequence Analysis

To identify the LAB isolates at the species level, total genomic DNA was extracted from overnight MRS broth cultures using the HigherPurity™ Bacterial Genomic DNA Isolation Kit (Canvax Biotech, Córdoba, Spain). The quality and concentration of DNA were assessed spectrophotometrically by measuring absorbance at 260 and 280 nm with a Shimadzu UV-VIS spectrophotometer (Shimadzu Corporation, Kyoto, Japan) [41]. The 16S rRNA gene was amplified using the LAB-specific primers LacbF (5′-TGCCTAATACATGCAAGT-3′) and LacbR (5′-CTTGTTACGACTTCACCC-3′), which have been successfully applied in previous studies for accurate LAB identification [40]. Each PCR reaction (20 µL) contained ~50 ng DNA template, 0.5 µM of each primer, and 8 µL of Red-Taq DNA Polymerase Master Mix (Canvax Biotech, Córdoba, Spain). Thermal cycling was conducted using a 2720 Thermal Cycler (Applied Biosystems, Waltham, MA, USA) under the following conditions: initial denaturation at 94 °C for 5 min; 35 cycles of 94 °C for 60 s, 50 °C for 45 s, and 72 °C for 2 min; and final extension at 72 °C for 5 min. Amplicons (~1200 bp) were visualized via electrophoresis on 1% agarose gel stained with SafeView™ nucleic acid dye (NBS Biologicals, Huntingdon, England) in 0.5× TBE buffer, run at 100 V for 60 min. Gels were imaged using a MiniBis Pro system (DNR Bio-Imaging Systems, Israel). Target bands were excised and purified using the Clean-Easy™ Agarose Gel Extraction Kit (Canvax Biotech). Sequencing was carried out by Microsynth Seqlab (Göttingen, Germany). The obtained sequences were compared with known sequences in the NCBI GenBank database using the BLAST v. 2.13.0 algorithm [42]. Identification was based on ≥97% sequence identity. Phylogenetic relationships between the LAB isolates and reference strains were inferred using the unweighted pair group method with arithmetic mean (UPGMA) implemented in CLC Genomics Workbench version 20.0 (QIAGEN Digital Insights). The 16S rRNA gene sequences of 39 LAB isolates were deposited in GenBank under accession numbers PQ482237–PQ482275 (submission: ID SUB14753976; 19 October 2024).

### 2.5. Molecular Identification of Yeast via ITS1–5.8S–ITS2 rRNA Gene Sequence Analysis

To identify the yeast isolates at the species level, single colonies were pre-cultured for 24 h on yeast malt agar (YMA) at 30 °C. Genomic DNA was extracted using the Higher-Purity™ Yeast Genomic DNA Isolation Kit (Canvax Biotech, Córdoba, Spain), following the standard protocol provided by the manufacturer. The concentration and purity of the DNA samples were assessed using a Shimadzu UV-VIS spectrophotometer (Shimadzu Corporation, Kyoto, Japan), with UV absorbance at 260/280 nm [41]. The internal transcribed spacer (ITS) region, encompassing ITS1–5.8S–ITS2, was selected as the barcode marker due to its high discriminatory power among yeast species [43]. PCR amplification was performed using the universal primers ITS4 (5′-TCCTCCGCTTATTGATATGC-3′) and ITS5 (5′-GGAAGTAAAAGTCGTAACAAGG-3′), synthesized by Metabion (Martinsried, Germany). Reactions were prepared in a final volume of 20 µL, including 1 µL of genomic DNA (~50 ng), 0.5 µM of each primer, and 8 µL of Red-Taq DNA Polymerase Master Mix (Canvax Biotech, Spain). Amplification was carried out in a 2720 Thermal Cycler (Applied Biosystems, Waltham, MA, USA) under the following conditions: initial denaturation at 95 °C for 10 min; 35 cycles of 94 °C for 1 min, 52 °C for 1 min, and 72 °C for 1 min; followed by a final extension at 72 °C for 7 min. PCR amplicons (~700 bp) were resolved on 1% agarose gel electrophoresis using SafeView™ DNA staining (NBS Biologicals, Huntingdon, England) in 0.5× TBE buffer, and visualized using the MiniBis Pro imaging system (DNR Bio-Imaging Systems, Israel) after electrophoresis at 100 V for 50 min. Amplicons were excised from the gel and purified using the Clean-Easy™ Agarose Gel Extraction Kit (Canvax Biotech, Córdoba, Spain). Bidirectional sequencing was performed by Microsynth Seqlab (Göttingen, Germany). The obtained ITS sequences were queried against the NCBI GenBank database using the BLASTn tool for species-level assignment based on ≥98% identity [42,44]. Phylogenetic analysis of representative isolates was conducted by clustering sequences with reference strains using the UPGMA algorithm in CLC Genomics Workbench v20.0 (QIAGEN Digital Insights). The ITS sequences from 29 yeast isolates were deposited in GenBank under accession numbers PQ482285–PQ482313 (submission number: SUB14801983; 19 October 2024).

### 2.6. Phylogenetic Analysis

The phylogenetic tree was constructed using the Unweighted Pair Group Method with Arithmetic Mean (UPGMA), a hierarchical clustering algorithm commonly employed to infer evolutionary relationships based on genetic distance matrices [45]. Sequence alignment and tree visualization were carried out using MEGAN version 11 (University of Tübingen, Germany) and ClustalW [46].

### 2.7. Statistical Analyses

All experimental measurements were performed in triplicate, and results are expressed as mean ± standard deviation (SD). Statistical evaluation of the data was conducted using one-way analysis of variance (ANOVA) to determine differences among sorghum hybrids. Post hoc comparisons of group means were carried out using Tukey’s honest significant difference (HSD) test to control for Type I error in multiple comparisons. A *p*-value of less than 0.05 was considered statistically significant. Significant differences among means are indicated by different superscript letters within each row or column. All statistical analyses were performed using XLSTAT software, version 2019.1.2 (Addinsoft, New York, NY, USA).

## 3. Results and Discussion

### 3.1. Nutritional Composition

The nutritional composition and antioxidant activity of three sorghum hybrids (Arabesk, Kalatur, and Zealandia) were evaluated, and the findings are summarized in Table 1.

The moisture content of cereals is an important quality indicator, since their shelf life depends largely on this parameter. Good-quality cereal grains must contain less than 14% moisture [47]. Buitimea-Cantúa et al., 2013 [48], reported moisture content in the range of 8.95–10.32% in different varieties of white sorghum grain. In the present study, all analyzed varieties showed higher values than the above, but still below the limit of 14%. Moisture content varied slightly among the hybrids, with Arabesk exhibiting the highest value (12.28%), followed by Kalatur (11.94%) and Zealandia (11.68%). Although the differences were minor, the lower moisture in Zealandia suggests slightly better stability during storage due to reduced risk of microbial growth.

According to previous research, the nutritional composition of sorghum grains is comparable to maize and rice in terms of protein, starch, and mineral content [16]. According to Tasie and Gebreyes (2020) [49], the proximate composition values such as moisture, ash, crude fat, crude fiber, crude protein, and carbohydrates of sorghum varied from 9.66 to 12.94, 1.12 to 2.29, 2.48 to 4.60, 2.17 to 8.59, 8.20 to 16.48, and 67.56 to 76.42, respectively. Another study explored sorghum flour obtained from 20 commercial hybrids grown in Argentina, with the average chemical composition of the samples amounting to 0.68% ash, 3.67% fat, 12.21% protein, 83.45% total carbohydrates, 79.77% starch (amylose 26.6%), and 34.9 mg of tannic acid per 100 g of flour [50].

The average crude protein content of sorghum is in the range of 10.04%, similar to wheat, teff, and other cereals [48]. In the present study, crude protein in both Kalatur (10.42 g/100 g) and Zealandia (10.33 g/100 g) was higher compared to Arabesk (9.37 g/100 g). This positions Kalatur and Zealandia as preferred candidates for protein-enriched, gluten-free food formulations. Chavan et al., 2017 [51], reported similar results for crude protein content: 9.15–11.56%.

Starch is the major constituent of sorghum, accounting for 56 to 75% of the total dry matter in the grain [52]. The three analyzed sorghum hybrids have relatively similar contents of total carbohydrates, ranging from 87.4 to 89.6 g/100 g. The total carbohydrate content was highest in Zealandia (89.6 g/100 g), with Arabesk and Kalatur showing slightly lower values (87.4 and 88.0 g/100 g, respectively). These results confirm the nutritionally beneficial high energy density of sorghum grains.

Crude fat content was comparable between Arabesk (4.97 g/100 g) and Zealandia (4.96 g/100 g), while Kalatur had a lower lipid content (3.84 g/100 g). The relatively high fat content in Arabesk and Zealandia may contribute to improved sensory characteristics such as mouthfeel and flavor, although attention must be paid to the potential risk of lipid oxidation during storage.

Total polyphenol content differed substantially across the hybrids. Zealandia exhibited the highest level (88.0 mg/100 g), followed by Kalatur (73.1 mg/100 g) and Arabesk (64.6 mg/100 g). These results are in line with the observed antioxidant activity, as measured with ORAC and HORAC assays. Zealandia demonstrated superior antioxidant potential, with ORAC and HORAC values of 67.1 µmol TE/g and 39.9 µmol GAE/g, respectively. Kalatur also showed substantial antioxidant capacity (61.7 and 35.6), while Arabesk had the lowest activity (45.5 and 28.7, respectively). Other studies also found that the antioxidant properties of sorghum had a strong correlation with the total phenolics of free and bound fractions, which was potentially attributed to the condensed tannins detected in the investigated sorghum hybrids [53,54,55,56].

Total flavonoid content (TFC) was not detected in any of the hybrids under the conditions of the performed analysis. On the other side, other authors reported the total flavonoid content of eight sorghum varieties ranging from 11.72 to 61.10 mg RE/100 g grain (DW) [57]. The results obtained in our study suggest that the antioxidant activity of the analyzed sorghum hybrids is mostly attributed to their polyphenol content, which could have a beneficial effect on sorghum-based functional foods. Another study also found that the correlation between TFC and antioxidant capacity was not so strong [58].

A previous study demonstrated that sorghum stalks contain many natural polyphenol compounds with high antioxidant activity and that their extracts can change bacterial morphology and internal structure, strongly inhibiting the growth of foodborne pathogens [59]. According to Mawouma et al. (2022) [60], red sorghum specifically exhibited the greatest amounts of total polyphenols (82.22 mg GAE/g DE), total flavonoids (23.82 mg CE/g DE), and total 3-deoxyanthocyanidin (9.06 mg/g DE). The most abundant phenolic compound was gallic acid, while the most frequent were chlorogenic and ferulic acids.

In the present study, analysis of free sugars revealed that the hybrid Kalatur contained the highest level of fructose (0.90 g/100 g), followed by Arabesk (0.73 g/100 g) and Zealandia (0.42 g/100 g). Arabesk had the highest amount of maltose (0.25 g/100 g), slightly exceeding Kalatur (0.19 g/100 g) and Zealandia (0.17 g/100 g). It was found that the fructose quantity in Arabesk (0.73 g/100 g) and in Zealandia (0.42 g/100 g) is relatively lower compared to Kalatur: 0.90 g/100 g. On the other hand, maltose content was relatively similar: 0.25 g/100 g in Arabesk, 0.19 g/100 g in Kalatur, and 0.17 g/100 g in Zealandia.

These variations in simple sugars may influence both taste perception and fermentability, which are relevant factors in cereal-based beverage production or sourdough fermentation. The Zealandia hybrid stood out across the parameters analyzed. It exhibited the lowest moisture, highest carbohydrate, highest protein and fat contents, richest polyphenol composition, and the most pronounced antioxidant activity. These findings underline its potential as a nutritionally superior hybrid suitable for functional food development. Kalatur also showed favorable characteristics, particularly in protein content and antioxidant activity, while Arabesk, although lower in some traits, displayed the highest maltose concentration, which may be of technological interest.

**Figure 1:**
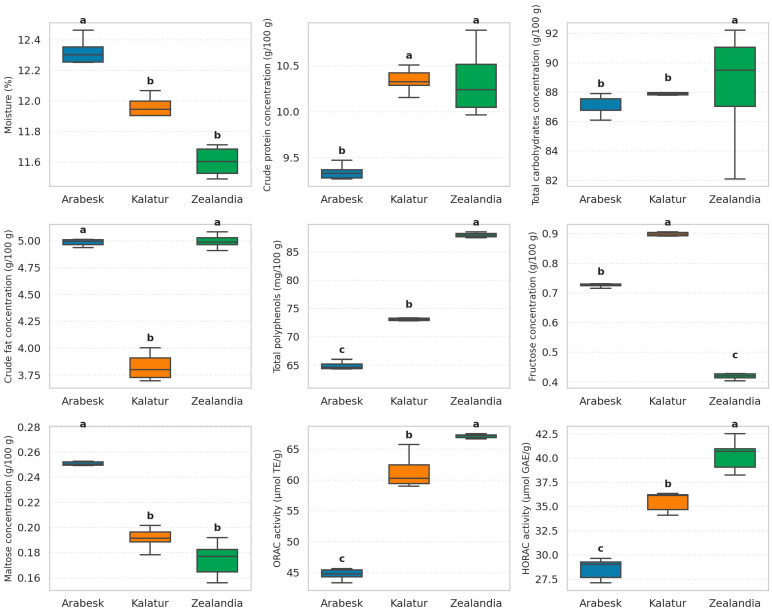
Boxplots illustrating the parameters that showed statistically significant differences among the three *Sorghum bicolor* hybrids (*Arabesk*, *Kalatur*, and *Zealandia*). Different letters (a, b, c) indicate pairwise differences that were statistically significant according to Tukey’s honest significant difference (HSD) test (*p* < 0.05).

Figure 1 shows a comparative overview of the analyzed key nutritional and functional parameters analyzed for the three sorghum hybrids Arabesk, Kalatur, and Zealandia.

The nutritional composition and antioxidant activity of the sorghum hybrids Arabesk, Kalatur, and Zealandia showed significant variation (Figure 1). Statistical analysis confirmed differences (*p* < 0.05) in moisture, protein, fat, polyphenol content, sugars, and antioxidant capacity. Zealandia had the lowest moisture and highest total carbohydrates, which suggests better storage stability. Both Zealandia and Kalatur had significantly higher protein content than Arabesk, enhancing their nutritional value for gluten-free diets. Arabesk and Zealandia were richer in crude fat, contributing to energy content and sensory attributes. Bioactive compounds in Zealandia contained the highest total polyphenols (88.0 mg/100 g) and exhibited the strongest antioxidant activity, as measured with ORAC and HORAC assays. These values were consistent with previous findings on pigmented sorghum varieties [61,62]. This hybrid demonstrates strong potential for use in functional foods with health-promoting properties.

Sugar composition also varied in Kalatur, which had the highest fructose, while Arabesk showed the highest maltose levels, which is important for fermentation applications. Zealandia showed superior quality across multiple parameters and may be considered the most promising candidate for food innovation and functional product development.

### 3.2. Fatty Acids

The fatty acid profiles of the sorghum hybrids Arabesk, Kalatur, and Zealandia revealed notable similarities, with differences mainly in the proportions of key unsaturated fatty acids (Table 2). The principal fatty acid components in the analyzed sorghum grains were palmitic (16:0), linoleic (18:2), and oleic (18:1) acids, which is consistent with previously published data [48,63,64,65,66,67]. Linoleic acid was the predominant fatty acid in all hybrids, accounting for 44.9% to 48.0% of total lipids, followed by oleic acid (31.9–38.2%) and palmitic acid (12.4–14.1%).

Kaplan et al. (2018) [68] obtained similar findings, reporting that major fatty acids in sorghum were linoleic acid (29.85–51.95%), oleic acid (30.62–49.73%), palmitic acid (10.96–22.02%), stearic acid (1.36–7.32%), and linolenic acid (0.58–5.41%). According to Zhang et al. (2019) [69], linoleic and oleic acids were the primary fatty acids in sorghum, accounting for more than 80% of the total fatty acids, while Hassan et al. (2017) [70] reported the highest fatty acid percentage for palmitic, stearic, and arachidic acid (13.75 ± 0.07%, 1.11 ± 0.09%, and 0.15 ± 0.03%, respectively). The saturated stearic acid (18:0) was found in the three presently analyzed hybrids at levels of 1.3–2.0%.

The balance between omega-6 and omega-9 fatty acids supports sorghum’s nutritional value as a source of essential polyunsaturated fatty acids (PUFAs). Zealandia had the highest content of PUFAs (49.4%), while Kalatur showed the highest monounsaturated fatty acid (MUFA) content (39.0%), which was mainly attributed to oleic acid. Palmitic acid (C16:0) was the dominant saturated fatty acid (12.4–14.1%), with minor contributions from stearic and arachidic acids. In all three varieties, PUFAs were higher than MUFAs.

Total saturated fatty acids (SFAs) were the lowest in Arabesk (14.8%) and slightly higher in Kalatur and Zealandia (15.4% and 15.3%, respectively). These hybrids exhibited a favorable lipid profile dominated by unsaturated fatty acids (79.0–87.9%), supporting their potential as functional ingredients in food products aimed at positively affecting heart health and LDL-cholesterol levels [71]. The particularly high unsaturated/saturated ratio in Kalatur and Zealandia further highlights their value in health-focused dietary applications. All three hybrids contained negligible amounts (<0.1%) of short- and medium-chain saturated fatty acids (C4:0–C15:1), which is consistent with typical cereal lipid patterns [72].

The total amount of unsaturated fatty acids in the analyzed sorghum hybrids varied from 79.0% in Arabesk to 87.9% in Zealandia. Unsaturated fatty acids are important for human nutrition, as they are major components of biological membranes and play a role in modulating the fluidity of membranes. Additionally, unsaturated fatty acids do not have cholesterogenic properties (unlike saturated fatty acids) and reduce the risk of thrombosis, due to the anti-aggregating activity of blood lipoprotein particles. Because of these features, unsaturated fatty acids are strongly recommended to lower the risk of atherosclerosis [66,71,72,73].

### 3.3. Organic Acids

The analysis of organic acids revealed notable differences among the three sorghum hybrids (Table 3). Quinic acid was the most abundant across all samples, with Kalatur showing the highest concentration (729.37 mg/100 g), followed by Arabesk (527.07 mg/100 g) and Zealandia (509.62 mg/100 g). Succinic acid was the second most prevalent, peaking in Kalatur (381.19 mg/100 g), indicating a potentially higher metabolic activity or microbial fermentation potential in this hybrid. Malic and tartaric acids were also present in considerable amounts, particularly in Kalatur, which showed elevated levels of both (92.83 and 181.02 mg/100 g, respectively), suggesting enhanced acidity and potential flavor contribution. Interestingly, acetic acid, which is typically associated with microbial metabolism, was higher in Arabesk and Kalatur but markedly lower in Zealandia. Kalatur stood out for its overall richness in organic acids, while Zealandia showed consistently lower levels across most acids, particularly acetic and succinic acids, which may influence its sensory profile and fermentation behavior. Fumaric and oxalic acids were detected in minor quantities in all samples, with the highest level of oxalic acid found in Kalatur (21.27 mg/100 g). These differences highlight the distinct biochemical signatures of the hybrids, which may affect their nutritional quality, taste, and suitability for fermentation or functional food development.

Quinic acid emerged as the dominant compound in all hybrids, particularly in Kalatur, which showed the highest concentration (729.37 mg/100 g). Quinic acid plays a role in plant defense and is also recognized for its antioxidant and antimicrobial properties, making Kalatur a promising candidate for functional food applications [74]. Succinic acid, a key intermediate in the tricarboxylic acid cycle, was also abundant, especially in Kaltur (381.19 mg/100 g), indicating a higher level of organic acid metabolism. Its presence has also been linked to improved microbial growth during fermentation and flavor development in cereal-based beverages.

### 3.4. Microbiological Analysis

The microbiological assessment of the sorghum hybrids Arabesk, Kalatur, and Zealandia revealed variation in the total viable counts, lactic acid bacteria, and yeast populations (Table 4).

The Zealandia hybrid exhibited the highest microbial counts across all tested groups, including LAB (5.3 × 10^3^ cfu/g), yeasts (11.2 × 10^3^ cfu/g), and TVC (18.4 × 10^3^ cfu/g). In contrast, Kalatur displayed the lowest microbial levels, with LAB and yeast counts of 2.8 × 10^3^ cfu/g and 7.4 × 10^3^ cfu/g, respectively. The differences noted may reflect the distinct grain surface characteristics, storage conditions, or cultivar-specific metabolic traits influencing microbial colonization. TVC was within the same levels as the LAB and yeast (10^3^ cfu/g), which could be attributed to the nystatin added in the culture media to suppress mold growth. The TVC levels in the analyzed sorghum grains were within the low values reported by Hall et al., 2011 [75], for wheat (2 to 9 log cfu/g) and wheat flour (1 to 6 log cfu/g). The presence of LAB and yeasts is considered beneficial, as these groups are associated with natural fermentation processes and potential probiotic effects [76,77].

The relatively low microbial loads (10^3^ cfu/g) are within the lower range reported for dry grains such as wheat and maize and reflect proper post-harvest storage and the natural microbiota of unprocessed sorghum. These levels are typical for cereals intended for fermentation starter selection rather than contaminated products.

Lactic acid bacteria and yeasts were isolated as pure cultures from the analyzed sorghum samples for further study. A total of 39 LAB isolates and 29 yeast isolates were recovered from the three hybrids. Kalatur yielded the highest number of isolates (16 LAB and 13 yeasts), indicating a richer and diverse microbial community. Arabesk and Zealandia each contributed to 11 and 12 LAB and 8 yeast isolates, respectively. The cultivable microbial diversity observed supports further investigation into strain functionality, particularly in the context of biopreservation, fermentation, and functional food development.

### 3.5. Phylogenetic Analysis of Lactic Acid Bacteria

**Figure 2:**
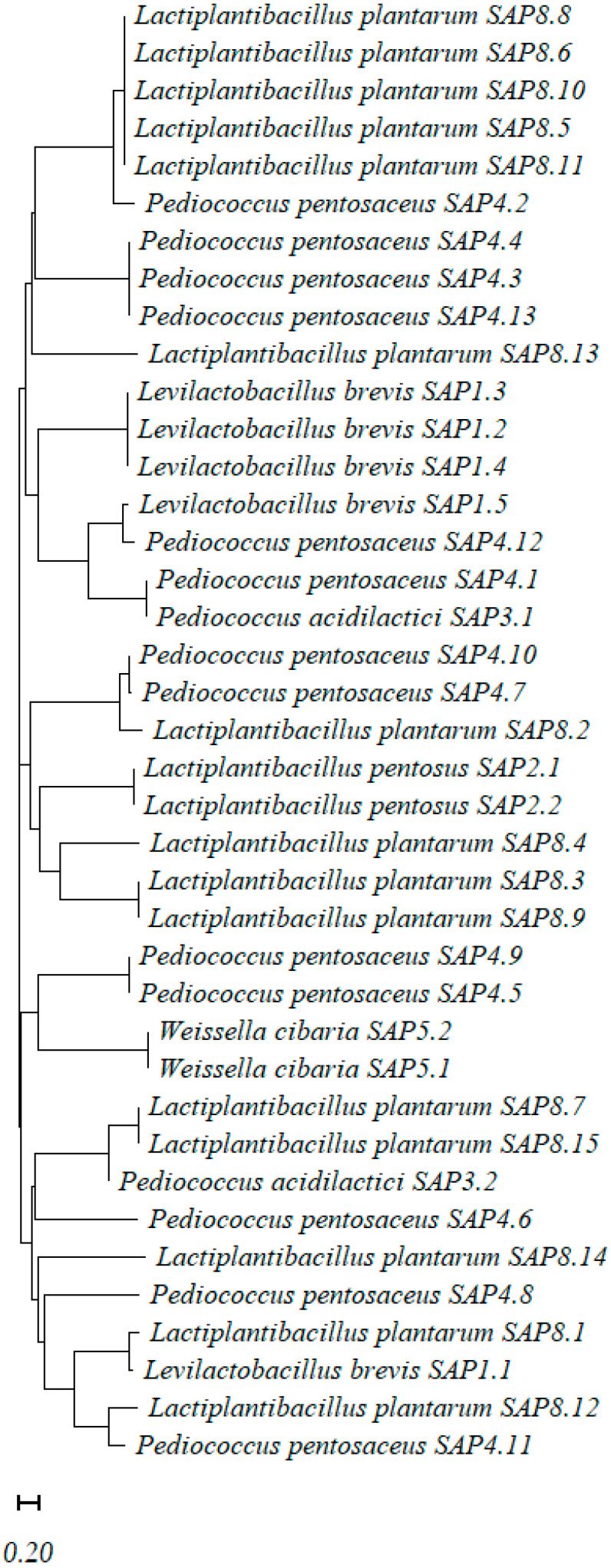
Phylogenetic tree of lactic acid bacteria isolated from sorghum hybrids Arabesk, Kalatur, and Zealandia, based on 16S rRNA gene sequences using UPGMA clustering.

The 39 LAB isolates recovered from the grains of *Sorghum bicolor* hybrids (Arabesk—11 strains; Kalatur—16 strains; and Zealandia—12 strains) were identified (Materials and Methods, *p*. 2.4, and the LAB sequences were deposited in GenBank under accession numbers PQ482237–PQ482275 (submission number: SUB14753976; 19 October 2024). Phylogenetic analysis was performed via UPGMA clustering of 16S rRNA gene sequences (Figure 2).

The largest cluster comprises *Lactiplantibacillus plantarum* strains, predominantly from Kalatur, highlighting its adaptability and competitive advantage in cereal-based fermentation systems. This species is widely recognized for its acid tolerance, metabolic versatility, and dominance in plant fermentations [76,78].

*Pediococcus pentosaceus* and *P. acidilactici* formed the second major group, especially present in Zealandia. These species are valued for their rapid acidification and production of antimicrobial compounds like bacteriocins, which help stabilize the fermentation [79]. A third cluster includes *Levilactobacillus brevis*, mainly from Arabesk. This heterofermentative species is associated with gas production and contributes to flavor complexity in cereal fermentations [76].

Two isolates from Zealandia were identified as *Weissella cibaria*, forming a distinct group. This species is gaining attention for its probiotic potential and production of exopolysaccharides, which can enhance texture and shelf life in fermented foods [80].

The species diversity across variants suggests that sorghum fermentation supports a rich and functionally diverse LAB community. Hybrid-specific strain composition likely reflects interactions between raw material composition and microbial ecology. These findings are consistent with previous studies on LAB diversity in traditional cereal fermentations [25,76,81].

### 3.6. Phylogenetic Analysis of Yeast Isolates

**Figure 3:**
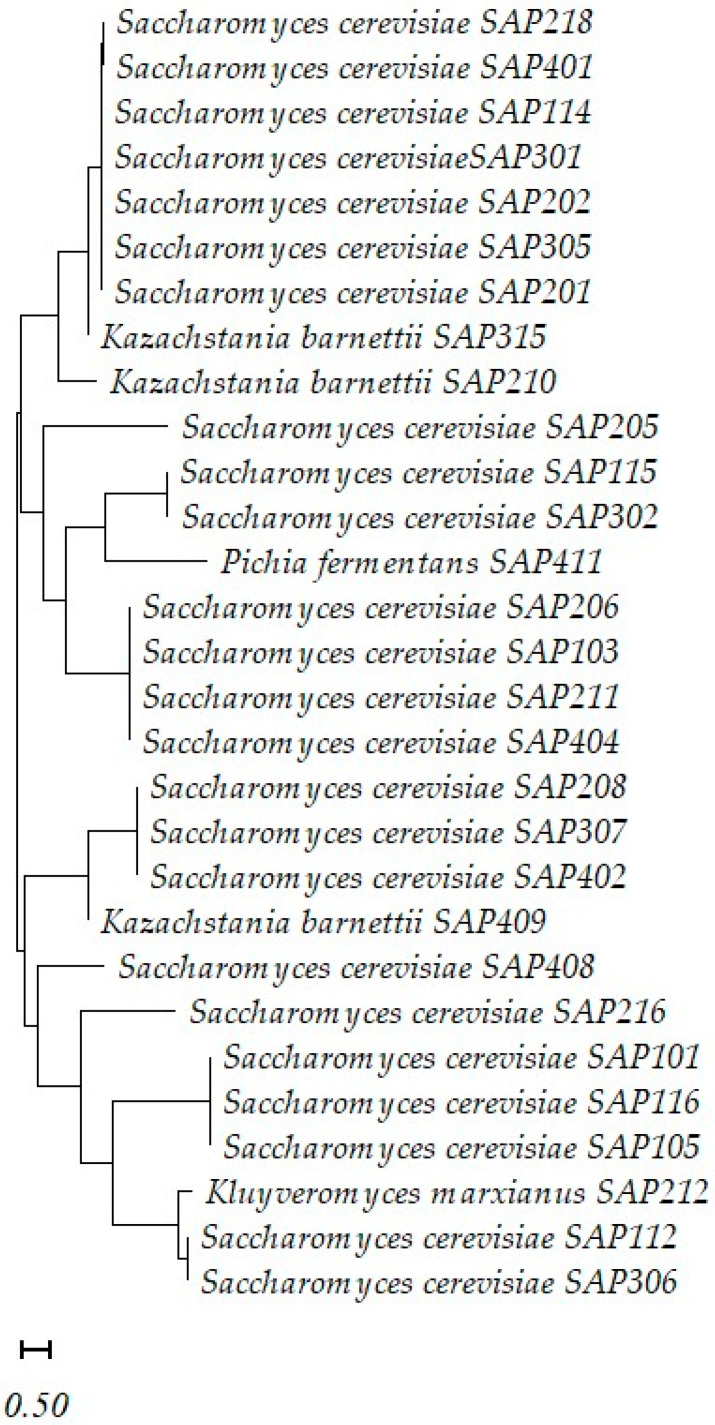
Phylogenetic tree of yeast strains isolated from grain of sorghum hybrids (based on ITS1–5.8S–ITS2 rRNA gene sequences).

The phylogenetic analysis of the 29 yeast isolates recovered from the three sorghum hybrids revealed the predominance of *Saccharomyces cerevisiae* across all three variants (Figure 3). This finding aligns with the well-established dominance of this species in spontaneous cereal fermentations due to its superior fermentative performance, ethanol tolerance, and adaptability to varying pH and sugar levels [31,82,83]. The majority of the isolates affiliated with *Saccharomyces cerevisiae* form multiple closely related sub-clusters, suggesting low intraspecies variability.

In addition to *S. cerevisiae*, the dendrogram reveals the presence of three non-Saccharomyces yeast species—*Kazachstania barnettii* (SAP210, SAP315, SAP409), *Pichia fermentans* (SAP411), and *Kluyveromyces marxianus* (SAP212). These species formed distinct clades, suggesting sufficient sequence divergence and supporting their phylogenetic independence. Their occurrence indicates a naturally diverse yeast consortium within the sorghum matrix, particularly in the Kalatur hybrid, which showed the highest diversity. Such species are known to contribute unique enzymatic profiles, flavor complexity, and potential technological functions [84,85].

Isolates from all three sorghum variants are distributed across the tree, indicating that clustering is not variant-specific. However, Kalatur contributed a slightly higher proportion of non-*Saccharomyces* strains, suggesting environmental or compositional influences on yeast community structure. The genetic diversity observed among the yeast strains underlines the complex and adaptable microbiota of sorghum fermentations and opens opportunities for the selection of yeast with tailored properties for improved nutritional and sensory profiles in sorghum-based foods.

The distribution of isolates across hybrids without strict clustering by variant suggests that the environmental conditions and indigenous microbiota, rather than the hybrid alone, influence yeast community composition. The coexistence of both fermentative and non-fermentative yeasts highlights the ecological interactions shaping sorghum fermentations, with implications for functional starter selection in future applications.

The microbial composition detected in the Bulgarian *Sorghum bicolor* hybrids showed both regional similarity and distinct ecological adaptation when compared with reports from Africa, Asia, and Europe. Similar to findings from traditional sorghum fermentations in Nigeria and India, *Lactiplantibacillus plantarum* and *Saccharomyces cerevisiae* were dominant representatives, indicating their widespread role in lactic and alcoholic fermentation processes. However, the consistent presence of *Weissella cibaria* and *L. plantarum* in Bulgarian hybrids suggests a stable, indigenous microbial consortium adapted to local agroclimatic conditions. These species are known for their probiotic potential and technological functions, such as the synthesis of organic acids, exopolysaccharides, and bacteriocins that contribute to the suppression of spoilage microorganisms and enhancement of nutritional and sensory quality. Furthermore, the metabolic activity of these strains supports the degradation of antinutritional factors (e.g., phytic acid and tannins) and the release of bioactive compounds that increase antioxidant capacity. Collectively, these findings highlight the unique microbiological and nutritional profile of Bulgarian sorghum hybrids, which combine high nutritional value with beneficial endogenous microbiota of functional importance.

The analyses conducted in our study provide critical insights that directly support the development of functional food products from *Sorghum bicolor* by offering a scientific basis for both nutritional value and microbial functionality. High polyphenol and antioxidant content, particularly in the Zealandia hybrid, indicates potential anti-inflammatory, anti-aging, and antioxidant effects, which are key attributes of functional foods targeting chronic disease prevention [86]. Balanced fatty acid profiles with high levels of polyunsaturated fatty acids (PUFAs like linoleic and oleic acids) support cardiovascular health and help reduce cholesterol, making sorghum suitable for heart-healthy product formulations [87,88,89]. Sorghum is naturally gluten-free and has a low GI due to its high fiber and resistant starch content, making it appropriate for individuals with celiac disease and type 2 diabetes [66,69,90]. Low sugar content and gluten-free nature make sorghum a suitable base for foods aimed at diabetics, celiac patients, and low-glycemic-index diets.

## 4. Conclusions

This study presents a comprehensive analysis of the nutritional composition, antioxidant capacity, organic acid profile, and microbial diversity associated with three sorghum hybrids cultivated in Bulgaria—Arabesk, Kalatur, and Zealandia. The results reveal significant inter-hybrid variability in macronutrients, bioactive compounds, and fatty acid profiles. Zealandia emerged as the most nutritionally robust hybrid, characterized by high carbohydrate and protein content, superior antioxidant activity, and a favorable ratio of polyunsaturated to saturated fatty acids. Microbiological assessments uncovered a diverse native microbiota dominated by lactic acid bacteria and yeasts, with *Levilactobacillus brevis*, *Lactiplantibacillus plantarum*, *Pediococcus* spp., and *Saccharomyces cerevisiae* as the most prevalent taxa. Phylogenetic analyses confirmed substantial strain-level heterogeneity and highlighted the presence of both fermentative and *non-Saccharomyces* yeasts with potential functional attributes. The microorganisms found on the grains contribute to the overall nutritional and biochemical profile of sorghum through their enzymatic activity and metabolite production.

These findings highlight the potential of sorghum as a nutrient-dense cereal with a rich indigenous microbiota suitable for use in functional foods and fermentation applications. The identified microbial isolates will be subjected to further studies for their potential use in controlled fermentations for the development of sorghum-based sourdough, beer, and bioethanol.

## Figures and Tables

**Table 1:** Nutritional composition, total polyphenols, and antioxidant activity of sorghum hybrids.

	Sorghum Hybrid		
Parameter	Arabesk	Kalatur	Zealandia
Moisture, %	12.28 ± 0.12 ᵃ	11.94 ± 0.08 ᵇ	11.68 ± 0.10 ᵇ
Crude protein, g/100 g	9.37 ± 0.07 ᵇ	10.42 ± 0.23 ᵃ	10.33 ± 0.30 ᵃ
Total carbohydrate, g/100 g	87.4 ± 0.67 ᵇ	88.0 ± 0.30 ᵇ	89.6 ± 4.26 ᵃ
Crude fat, g/100 g	4.97 ± 0.04 ᵃ	3.84 ± 0.12 ᵇ	4.96 ± 0.08 ᵃ
Total polyphenols, mg/100 g	64.6 ± 0.9 ᶜ	73.1 ± 0.6 ᵇ	88.0 ± 0.6 ᵃ
Total flavonoids, mg/100 g	n.d. *	n.d.	n.d.
Free sugars, g/100 g			
Fructose	0.73 ± 0.01 ᵇ	0.90 ± 0.02 ᵃ	0.42 ± 0.02 ᶜ
Maltose	0.25 ± 0.01 ᵃ	0.19 ± 0.01 ᵇ	0.17 ± 0.01 ᵇ
Antioxidant activity			
ORAC, µmol TE/g	45.5 ± 1.4 ᶜ	61.7 ± 2.6 ᵇ	67.1 ± 0.3 ᵃ
HORAC, µmol GAE/g	28.7 ± 1.2 ᶜ	35.6 ± 2.1 ᵇ	39.9 ± 1.4 ᵃ

Data are presented as the mean ± SD. Different superscript letters within a row indicate statistically significant differences among hybrids according to Tukey’s HSD test (*p* < 0.05); * n.d.—not detected.

**Table 2:** Fatty acid profiles of sorghum hybrids Arabesk, Kalatur, and Zealandia.

		Sorghum Hybrids		
Fatty Acids. %		Arabesk	Kalatur	Zealandia
C 4:0	Butyric	<0.1	<0.1	<0.1
C 6:0	Caproic	<0.1	<0.1	<0.1
C 8:0	Caprylic	<0.1	<0.1	<0.1
C 10:0	Capric	<0.1	<0.1	<0.1
C 12:0	Lauric	<0.1	<0.1	<0.1
C 14:0	Myristic	<0.1	<0.1	<0.1
C 15:0	Pentadecanoic	<0.1	<0.1	<0.1
C 15:1	10(Z)-Pentadecenoic	<0.1	<0.1	<0.1
C 16:0	Palmitic	12.4	14.1	13.5
C 16:1	Palmitoleic	0.5	0.6	0.6
C 17:0	Margaric	0.2	<0.1	0.2
C 17:1	Heptadecenoic	<0.1	<0.1	<0.1
C 18:0	Stearic	2.0	1.3	1.3
C 18:1	Oleic	31.9	38.2	37.5
C 18:2 n-6	Linoleic	44.9	46.7	48.0
C 18:3 n-3	Linolenic	1.5	1.6	1.4
C 20:0	Arachidic	0.2	<0.1	0.3
C 20:1	Gadoleic	0.2	0.2	0.3
C 22:0	Behenic	<0.1	<0.1	<0.1
C 22:1	Erucic	<0.1	<0.1	0.1
C 24:0	Lignoceric	<0.1	<0.1	<0.1
Saturated fatty acids		14.8	15.4	15.3
Unsaturated fatty acid		79.0	87.3	87.9
Monounsaturated fatty acid		32.6	39.0	38.5
Polyunsaturated fatty acid		46.2	48.3	49.4

**Table 3:** Organic acid composition of sorghum hybrids.

	Organic Acids, mg/100 g						
Hybrid	Oxalic Acid	Tartaric Acid	Quinic Acid	Malic Acid	Acetic Acid	Succinic Acid	Fumaric Acid
Arabesk	10.91 ± 0.10 ^a^	156.26 ± 1.40 ^b^	527.07 ± 10.24 ^b^	68.42 ± 2.23 ^a^	66.83 ± 1.38 ^c^	269.37 ± 2.00 ^b^	1.11 ± 0.01 ^b^
Kalatur	21.27 ± 0.03 ^c^	181.02 ± 2.68 ^c^	729.37 ± 1.81 ^c^	92.83 ± 0.21 ^c^	63.19 ± 1.52 ^b^	381.19 ± 1.44 ^c^	1.03 ± 0.03 ^b^
Zealandia	11.80 ± 0.48 ^b^	138.00 ± 2.50 ^a^	509.62 ± 9.44 ^a^	71.24 ± 1.51 ^b^	33.47 ± 0.30 ^a^	221.52 ± 2.29 ^a^	0.79 ± 0.02 ^a^

Data are presented as the mean ± SD. Different superscript letters within a column indicate statistically significant differences among hybrids according to Tukey’s HSD test (*p* < 0.05).

**Table 4:** Microbiological assessment of sorghum grains.

	Sorghum Hybrid		
Parameter	Arabesk	Kalatur	Zealandia
LAB, cfu/g	4.1 × 10^3^	2.8 × 10^3^	5.3 × 10^3^
Yeast, cfu/g	9.7 × 10^3^	7.4 × 10^3^	11.2 × 10^3^
TVC, cfu/g	14.4 × 10^3^	10.9 × 10^3^	18.4 × 10^3^

## Data Availability

The original contributions presented in this study are included in the article. Further inquiries can be directed to the corresponding author. The 16S rRNA gene sequences of LAB isolates were deposited in GenBank under accession numbers PQ482237–PQ482275 (submission ID: SUB14753976; October 19, 2024) at https://www.ncbi.nlm.nih.gov/nuccore/?term=PQ482237:PQ482275. The ITS sequences of yeast isolates were deposited in GenBank under accession numbers PQ482285–PQ482313 (submission ID: SUB14801983; 19 October 2024) at https://www.ncbi.nlm.nih.gov/nuccore/?term=PQ482285:PQ482313 (accessed on 19 October 2024).
